# Supplementary material for: Combined analysis of transposable elements and structural variation in maize genomes reveals genome contraction outpaces expansion
Source: PLoS Genet. 2023 Dec 22;19(12):e1011086. doi: 10.1371/journal.pgen.1011086 (PMC10773942; doi:10.1371/journal.pgen.1011086)
Supplement: Attachment — Submitted filename: Editor_review_Munashige_et_al.pdf [file pgen.1011086.s012.pdf]

Dear authors,
Thank you for submitting your work to PLOS Genetics. The manuscript from Munashige et al. contributes to the field by addressing a long-standing question on determining whether genome expanses brought by transposable elements (TEs) can be counter-balanced by other molecular processes. The rationale of the paper is clearly explained, and highly relevant for plant genome evolution.

The two reviewers point the interest of the biological question addressed as well as the adequation of the methodology set up to answer it. They also raise some specific points which are relevant to improve the manuscript.

In addition to the comments of the reviewers, I have myself some points that I would like the authors to address, in particular on the methodology used.
Although the overall rationale of the methodology (*i.e.*, detecting structural variants between maize lines from whole genome sequence assembly comparisons, and crossing the results with TE positions from annotations) is relevant, I have concerns on the extent to which the combination of panEDTA and AnchorWave information allows to accurately answer the question addressed.

In particular, the authors observe a large number of structural variants (SVs) encompassing several TE copies, and conclude that these variants correspond to deletions. But couldn't they also correspond to nested transposable elements structures, where TEs are piled into one another? If the oldest elements (at the bottom of the pile) are highly degraded, there is a possibility that a homology could be found on one side of the pile, but not on the other, leading to the prediction of two different elements at the two borders of the variant. This would lead to prediction of a deletion, where it is actually a block of insertions. Nested cases like this have been described in Liu et al. 2007 – PNAS and checking how these cases are resolved by the methodology used is crucial to make sure that the methodology sustains the conclusions found. More largely, a major point would be to determine whether the structural variants encompassing several TEs correspond to nested cases, or to series of independent insertions not piled within one another.

The authors provide a schematic view of the analysis of one region (Figure 1), but it is difficult to understand to what extent this example is representative of all cases. In particular, regions that have been previously characterized such as the *Bronze* region (Wang and Dooner, 2006 – PNAS) are not presented. Comparing the results found by the procedure used (positions of TE annotations predicted by panEDTA, position of SVs detected by AnchorWave) to these of previous analyses in well annotated regions is an important benchmark.
Also, to claim that the underlying mechanism is deletion, the authors should provide an analysis of the breakpoints. In particular, stretches of microhomology have been observed for presence/absence variants in several plant species including maize. Are similar patterns observed? What is the size range of the stretches and does it fit this observed in previous reports? (see for instance Darracq et al., 2014 – BMC Genomics; Vitte and Bennetzen, 2006 – PNAS)

All the analyses presented in Figure 3 are based on all types of TE variants. But these include both insertions of single elements, and variants including several TEs. How are the latest impacting the results? If the aim of this series of results is to compare the polymorphism of insertions among families and superfamilies, the analysis should be restricted to cases where TE=SV, without taking into account SVs encompassing several TE copies, which could correspond to other processes than insertion.
It would be nice to have a chromosomal representation of variants across the B73 chromosomes, to have an idea of local densities. A color code could be used to highlight the distribution of the different variant cases.

p.15, l.309-311: The large variants could also correspond to large insertions, rather than to large deletions. Could the authors state how they can discriminate the two?

p.16, l.316-319: Describe how many regions that corresponds to, what is there size distribution and where they are located in the genome. Move paragraph "The size of the regions…B73 genome" upper and give more details on size distribution and chromosomal locations.

The analysis of the SNP-depleted regions allows to find 139 cases of 'TE=SV' among a total of 690 cases (p.17, l.354). This makes about 20% of the cases. But in the rest of the genome, the authors find a percentage of about 10%. Could the authors comment on this? This suggests that non 'TE=SV'

variants are more often occurring in the SNP-containing regions of the genome. Are SV sizes larger in the SNP-containing regions? This could fit with a larger abundance of nested TE in the more divergent regions, and could point to presence/absence of these nests rather than to large deletions.

As pointed reviewer #1, it is very important for the community to have access to all variants detected. This should be supplied as supplementary material.

There are also minor points that should be corrected in the manuscript:

p.2 l.32: Precise if referring to numbers of events, or number of base pairs
p.3 l.40: Add drift
p.4 l. 63: Actually, a large number of studies have analyzed the effect of deletions (see for instance Devos et aL;, 2022 - Genome Research and Vitte and Bennetzen 2006 – PNAS). The models did take into account the deletion process (see for instance Vitte and Panaud, 2005 – CGR). But due to limited quality of the genome assemblies at the time, these studies were limited to analysis of internal deletions located within TEs, and could not take into account large deletions. This is what is accessible now that high quality genome assemblies are available for several individuals.
p.4 l. 72. The authors should acknowledge that these pioneer studies gave important insights on TE-related maize genome structure and evolution, in particular on the importance of TE insertions and recombination to generate the TE landscape observed in different maize inbred lines. They were nevertheless limited in the amount of genomic sequence analyzed, and therefore whole genome assemblies now allow for accurate quantification of the processes involved.
p.6 l. 124: Move reference after 'AnchorWave'.
p.6 l. 128: What is the range observed?
p.7 l. 147: Change 'inserted sequence' to 'SV'
p.8 l. 157: Are the results in line with the genetic distance between B73 and the different NAM founders?
p.8 l.165: Change 'low polymorphic rate' to 'low rate of polymorphic cases'
p.8 l.168: "… estimates": add reference
p.9, l. 184: So what was the overlap between non syntenic genes and TEs in this study? Why were these 'genes' not considered as TEs?
p.9, l.188: "… TE mobilization": add reference
p.10, l/214: Superfamilies full name should be indicated in italics
p.11 l.218-219: Isn't this expected by the cut and paste mechanism?
p.11 l.229: Change 'elements within a family' by 'copies within a TE family'
p.14, l.279: Please precise the size range observed
p.14, l.281: "SV sequences that have partially (>95%) overlapping TE sequences": does this mean that 95% of the SV length is made of TE sequence or that 95% of the TE is within the SV? From the examples, it seems that it is the second case. Please clarify. Could cases of SV located within larger TEs correspond to misannotated insertion within the TE rather than to deletions? To what extent could such insertions be missed in the annotation?
p.15, l. 298: On average, 85% of the SVs is annotated as TE bases. This proportion is similar to this of TE sequences within the B73 genome. Could the authors comment on this? This could suggest that SV occur randomly in the genome rather than that TEs are major contributors to SVs.
p.17, l.334: How was the 100-fold threshold chosen?
p.17, l.339: Is this excepted considering the pedigree of the B73 inbred line? Where are these regions located? Are they shared between the NAM lines where they are found?
p.17, l. 343: 'the SNP depleted regions': do they correspond to the 213 or to the subset presented above?
p.18, l.356: why 224 and not 139?
p.18, l.365: 'these regions': please precise which ones
p.18, l.376: 'sequence similarity': do the authors mean 'sequence identity'?
p.21, l.428: Please cite previous work, such as this of Wang and Dooner, 2006 – PNAS
p.22, l.454, l.457, l.458: please check typos and missing information
p.23, l.478: The authors could have proof of TE-related ectopic recombination using their data: what is the number of cases where the same TE family is found at the two borders, with breakpoint at the same site?
p.23, l.480: The authors should also discuss the presence of deletions within the TE sequences. What is their proportion in the analyzed dataset?
p.24, l.507: Were long terminal repeat regions really removed?

p.26, l.537: Please precise the fraction overlapping TE annotations
p.26, l.554-556: For SVs that were found between B73 and several maize lines, were the breakpoints found always the same at the base level? This would help deciphering the accuracy of breakpoint prediction by AnchorWave.

Figure 1:
Precise the amount of overlap required for ambiguous cases
Figure 1 B: Add colors showing the amount corresponding to the different features (TE, gene syntenic, gene non syntenic

Figure 3A: Please add a statistical test
Figure 3B: Please state how many families the >20 copies threshold leads to
Figure 3C: The pattern observed is also likely impacted by the age of the copies. How does the pattern correlate with age of the copies? Is the age of the small families similar to this of families with larger copy number?
