# Supplementary material for: Combined analysis of transposable elements and structural variation in maize genomes reveals genome contraction outpaces expansion
Source: PLoS Genet. 2023 Dec 22;19(12):e1011086. doi: 10.1371/journal.pgen.1011086 (PMC10773942; doi:10.1371/journal.pgen.1011086)
Supplement: Attachment — Submitted filename: ResponseToReviewers_Munasinghe_etal_10.04.23.pdf [file pgen.1011086.s013.pdf]

Response to reviewers - Munasinghe et al.

Overall Response: We very much appreciate the comments of the editor and reviewers. Many common themes emerged and these reflect lack of clarity in the presentation and interpretation of results in our initial draft. We have worked to revise the manuscript and provide detailed responses below. We wish to highlight several main changes.

1. The logic for insertions and deletions is quite complex when applied genome-wide since this includes many haplotypes with long divergence times. It is quite difficult to really differentiate between nested insertions and large deletion events, and it is even more difficult to construct a logic flow or pipeline that can do this effectively. Consequently, we are only applying this logic and framework to the SNP depleted regions. These are recently diverged haplotypes, and, therefore, they are unlikely to have new nested insertions. These regions should also have young TE insertions that would be much better annotated than old, decaying elements (the natural decay of TEs over time is something that makes differentiating between nested elements and large insertions difficult)
2. It occurred to us that the Data availability statement may not have been presented with the initial manuscript. This information was submitted through the PloS Genetics dashboard, but we realized that this was not included in the manuscript text. This may have added to the confusion about what data was made publicly available with this manuscript. We apologize for this, and we have added this to the manuscript with clear descriptions on the data products.

Editor comments:

Although the overall rationale of the methodology (i.e., detecting structural variants between maize lines from whole genome sequence assembly comparisons, and crossing the results with TE positions from annotations) is relevant, I have concerns on the extent to which the combination of panEDTA and AnchorWave information allows to accurately answer the question addressed.

We certainly understand and appreciate this concern. Annotating TEs and identifying TE variation is highly complex, and there are often exceptions that complicate genome-wide analyses. We have worked to provide further support for the approaches that we have implemented and have also tried to add more context and potential caveats to the text to explain potential issues.

In particular, the authors observe a large number of structural variants (SVs) encompassing several TE copies, and conclude that these variants correspond to deletions. But couldn't they also correspond to nested transposable elements structures, where TEs are piled into one another? If the oldest elements (at the bottom of the pile) are highly degraded, there is a possibility that a homology could be found on one side of the pile, but not on the other, leading to the prediction of two different elements at the two borders of the variant. This would lead to prediction of a deletion, where it is actually a block of insertions.
Nested cases like this have been described in Liu et al. 2007 – PNAS and checking how these cases are resolved by the methodology used is crucial to make sure that the methodology sustains the conclusions found.

More largely, a major point would be to determine whether the structural variants encompassing several TEs correspond to nested cases, or to series of independent insertions not piled within one another.

This is a valid point. Nested TE insertions are quite common in maize and have been described by several groups. We have added language to specify that the MultiTE SVs could include deletions as well as examples of nested TEs. There are several main ways that we have worked to address this issue.

First, in the manuscript, we have tried to be careful to apply the logic on insertions / deletions primarily to the SNP-depleted regions of the genome. These are regions that have been separated for a limited number of generations and, therefore, have had little time to accumulate multiple independent TE insertions at a single region. So, recent nested insertions are quite unlikely in these regions. This gives us further confidence that the Multi TE SV events in these regions are deletions rather than nested insertions.

Second, we have done an analysis to assess the frequency of TE annotations that span the edge of an SV edge. If there is a single TE annotation that overlaps at least 50bp of the edge of the SV and 50bp of the flanking region, it suggests that there was a single TE at this region and a subsequent deletion removed a portion of the TE. This analysis was applied to the SVs within the SNP depleted regions, and we found that many (74.6%) of the Multi TE SVs in these regions have at least one edge that overlaps part of a TE (at least 50bp of the TE is in the SV and at least 50bp is outside). This provides additional evidence that many of these Multi TE SV events are likely deletions rather than recent nested insertions.

Third, we searched for potential signatures of LTRs or TIRs in the edges of the Multi TE-SV events. We did this by blasting the left edge of the SV against the right edge of the same SV. For LTRs, we took any SV 600bp. We then took the left 100bp and blasted it against the right 500bp. The longest alignment (>50bp) in the same orientation meant that SV was classified as a putative LTR insertion. For TIRs, we took any SV 100bp. We then took the left 50bp and blasted it against the right 50bp. The longest alignment (of any size) in the reverse orientation classified that SV as a putative TIR insertion. For any SVs that belong to both categories, we retain the classification derived from the longest alignment. Untested SVs were defined as an SV that was either (a) < 100bp in length (as it could not be tested as a TIR in our analysis) or (b) SVs between 100bp and 600bp long that were not classified as TIRs (as they were too short to be tested as an LTR). All remaining SVs were classified as putative deletion event (i.e., there was no blast hit). In the SNP depleted regions, we found that 84.5% of the Multi TE SV events have no evidence of either LTRs or TIRs (i.e., there were no blast hits between the edges of the SV). We find that 6.9% have evidence of TIRs (i.e., there is a blast alignment >50 bp in the and another 6.9% have evidence of LTRs. However, it is worth noting that we only required 10bp of direct or inverted homology so these might be over-estimates, especially for the LTRs. Genome wide, we see very similar numbers, 85.4% have no homology between the 5' end and the 3' end, while 3.9% and 6.7% have potential evidence for LTRs or TIRs (respectively).

Together, these approaches suggest that while nested TEs are likely quite common, they are likely not accounting for the Multi TE SV events in the SNP depleted regions.

The authors provide a schematic view of the analysis of one region (Figure 1), but it is difficult to understand to what extent this example is representative of all cases. In particular, regions that have been previously characterized such as the Bronze region (Wang and Dooner, 2006 – PNAS) are not presented. Comparing the results found by the procedure used (positions of TE annotations predicted by panEDTA, position of SVs detected by AnchorWave) to these of previous analyses in well annotated regions is an important benchmark.

It is worth noting that the schematic in Figure 1 is not actually showing any particular region of the genome. This is simply a schematic that illustrates the output types from Anchorwave and how features would overlap and be classified. The actual data is represented in panels B-E of this figure. The reviewer is correct that the *bz* genomic regions has been previously reported and is a useful locus. However, prior work on this locus utilized very different TE annotation approaches and while it included B73 it did not include other NAM genomes meaning that the prior work would not be directly comparable to the current study. We would highlight that the R Shiny app mentioned in the Data Availability section allows the reviewer (or any reader) to visualize any region of the genome (https://mmunasin.shinyapps.io/nam_sv/) and compare it to their expectations.

Also, to claim that the underlying mechanism is deletion, the authors should provide an analysis of the breakpoints. In particular, stretches of microhomology have been observed for presence/absence variants in several plant species including maize. Are similar patterns observed? What is the size range of the stretches and does it fit this observed in previous reports? (see for instance Darracq et al., 2014 – BMC Genomics; Vitte and Bennetzen, 2006 – PNAS)

This is an interesting point. Microhomology mediated end joining (MMEJ) is certainly a potential mechanism for deletions. However, this is not necessarily a hallmark of all deletions. Other DNA repair mechanisms can repair breaks and result in deletions as well. We did perform an analysis of TE features that are partially overlapping an SV which would suggest a partial deletion of a larger feature that supports many of the deletions (see earlier response for details).

All the analyses presented in Figure 3 are based on all types of TE variants. But these include both insertions of single elements, and variants including several TEs. How are the latest impacting the results? If the aim of this series of results is to compare the polymorphism of insertions among families and superfamilies, the analysis should be restricted to cases where TE=SV, without taking into account SVs encompassing several TE copies, which could correspond to other processes than insertion.

The aim in this figure / analysis was not necessarily to compare only insertion polymorphisms. Instead the goal was simply to ask if certain types of TEs are more likely to be polymorphic (via either insertion or deletion). The editor is correct that this could include both TE=SV events as well as larger SVs that include multiple TEs. We did not add an additional analysis solely focused on TE-SV events for this.

It would be nice to have a chromosomal representation of variants across the B73 chromosomes, to have an idea of local densities. A color code could be used to highlight the distribution of the different variant cases.

We are not entirely certain of what the reviewer is looking for in this comment. We agree that it would be possible to generate a chromosomal representation in which we show either the bp or # of events for sliding windows (maybe 500Mb) along the chromosomes. However, these could be quite complex and could be visualized in many ways. For example, if we show the B73 genome is the reviewer interested in comparing to one of the other NAM lines or summing the variants relative to all NAM lines. Is the reviewer more interested in polymorphic TEs, or types of SVs events? We are open to making this plot if there is a compelling reason and clear use for the plot.

p.15, l.309-311: The large variants could also correspond to large insertions, rather than to large deletions. Could the authors state how they can discriminate the two?

Good point - this interpretation has been removed from this region. We apply a different interpretation at this stage of the manuscript to better reflect the ambiguous nature of some of these events based on a genome wide analysis. We also mention above additional analyses we did in order to gain additional evidence that the Multi TE SV events in SNP depleted regions likely represent deletions rather than netsed insertions.

p.16, l.316-319: Describe how many regions that corresponds to, what is there size distribution and where they are located in the genome. Move paragraph "The size of the regions…B73 genome" upper and give more details on size distribution and chromosomal locations.

Good suggestion. We had this detail in the supplemental table but have now added these details to the text as follows. "There were a total of 213 of these SNP depleted regions that were identified based on comparisons of B73 to all of the other 25 NAM inbred parents (Table S2). The size of the regions was quite variable (min=2Mb, max=25.5Mb, median=3.1Mb) (Figure S4). While many (26 out of 213) of the regions were only 2Mb in length in B73, there were also 23 SNP depleted regions that were at least 10Mb in length. These 213 SNP depleted regions accounted for approximately 1,048Mb cumulative base pairs of the B73 genome. SNP depleted regions are found across all 10 chromosomes. There were no SNP depleted regions identified in comparisons fo B73 and four of the NAM genomes (CML69,CML247, CML277, and NC350). In contrast, > 20 regions were identified between B73 and each of B97 (N = 22), Ky21 (N = 27), MS71 (N = 21), Oh7B (N = 25), and Oh43 (N = 31)."

The analysis of the SNP-depleted regions allows to find 139 cases of 'TE=SV' among a total of 690 cases (p.17, l.354). This makes about 20% of the cases. But in the rest of the genome, the authors find a percentage of about 10%. Could the authors comment on this? This suggests that non 'TE=SV' variants are more often occurring in the SNP-containing regions of the genome. Are SV sizes larger in the SNP-containing regions? This could fit with a larger abundance of nested TE in the more divergent regions, and could point to presence/absence of these nests rather than to large deletions.

We are not sure where the reviewer estimates of 10% and 20% for SNP-depleted and genome-wide frequencies of TE=SV events are derived from. We report that 20.14% (139/690 – Page 19, line 383) of the B73 SVs within the SNP depleted regions and 26.8% (Page 15 line 303) of the B73 genome-wide SVs are TE=SV events. This means that there are actually more TE=SV events genome-wide than in the SNP-depleted regions. There is a difference in the median size of SVs genome-wide (median=1,557bp) compared to the SNP depleted regions (median=874bp), and this could be due to more complex nested insertions genome-wide or could be due to other factors as well.

As pointed reviewer #1, it is very important for the community to have access to all variants detected. This should be supplied as supplementary material.

We agree with the reviewer that several of the datasets referenced in our manuscript would be of use to the community and should, therefore, be made publicly available. We would like to note, however, that some of these datasets are already available. We used TE annotations generated using panEDTA (developed by Ou et al. in Review). While this manuscript is currently under review, the TE annotation files are publicly available on maizeGDB (https://maizegdb.org/NAM_project). We performed filtering on the full list of annotated TEs to generate a reduced set of TE annotations that we had much higher confidence in. This set of TE annotations, as well as their status as either shared/polymorphic/ambiguous, has been uploaded to Dryad as part of the Data Availability of this manuscript. It occurred to us that it may not be clear from our manuscript that this full set of annotations is publicly available, and we have added a sentence to the methods highlighting this.

“These annotations are publicly available for download through MaizeGDB (https://maizegdb.org/NAM_project).”

Similarly, while we provided the AnchorWave MAFs and GVCFs which we parsed to call structural variants, filtering would have to be applied in order to recover the structural variants we use. Consequently, we now provide our ‘summarised’ output which distinctly lists the locations of all structural variants (these are in bed format as there is no strand/score information) and have uploaded these files to Dryad.

In sum, all data involved in this manuscript is either directly provided via Dryad or is publicly available on MaizeGDB. It occurred to us that our Data Availability section may not have been shared as part of the manuscript submission. We have now explicitly added that section to the end of our manuscript.

There are also minor points that should be corrected in the manuscript:
p.2 l.32: Precise if referring to numbers of events, or number of base pairs

We have edited this section to clarify.

p.3 l.40: Add drift

Good point, we have amended this sentence to include drift. "Mutation followed by selection, in addition to random genetic drift, is the basis for evolution and drives diversification."

p.4 l. 63: Actually, a large number of studies have analyzed the effect of deletions (see for instance Devos et aL;, 2022 - Genome Research and Vitte and Bennetzen 2006 – PNAS). The models did take into account the deletion process (see for instance Vitte and Panaud, 2005 – CGR). But due to limited quality of the genome assemblies at the time, these studies were limited to analysis of internal deletions located within TEs, and could not take into account large deletions. This is what is accessible now that high quality genome assemblies are available for several individuals.

We have written this section to now read:
"On-going deletion events in many plant genomes can counteract genome size expansion caused by TE accumulation (8-13). Several studies have explored the influence of deletion events on genome size variation (14-16 [for context - Ma et al. 2004, Vitte and Panaud 2005, Vitte and Bennetzen 2006]), and they have been instrumental in understanding how TEs shape genome evolution. However, these studies were limited in scope due to technological limitations.

p.4 l. 72. The authors should acknowledge that these pioneer studies gave important insights on TE-related maize genome structure and evolution, in particular on the importance of TE insertions and recombination to generate the TE landscape observed in different maize inbred lines. They were nevertheless limited in the amount of genomic sequence analyzed, and therefore whole genome assemblies now allow for accurate quantification of the processes involved.

That is a fair point, and the revised text now addresses this.

p.6 l. 124: Move reference after 'AnchorWave'.

Done.

p.6 l. 128: What is the range observed?

We have amended this sentence to now reads: "Across all pairwise comparisons between B73 and all other NAM lines, 603% of the B73 genome was alignable (min=57.6%, max=63.6%), 36.5% was inserted sequence in B73 (min=33.1%, max=38.8%), and 3.2% was unalignable (min=2.7%,max=3.8%) when compared to the other genomes (Figure 1B,S1).

p.7 l. 147: Change 'inserted sequence' to 'SV'

Done

p.8 l. 157: Are the results in line with the genetic distance between B73 and the different NAM founders?

Done

p.8 l.165: Change 'low polymorphic rate' to 'low rate of polymorphic cases'

Done

p.8 l.168: "... estimates": add reference

Done

p.9, l. 184: So what was the overlap between non syntenic genes and TEs in this study? Why were these 'genes' not considered as TEs?

We used the classification of syntenic vs non-syntenic genes determined by the NAM paper (Hufford et al. 2021 Science). In this study, they applied several tools to remove putative genes that encoded TE related sequences. This results in relatively little overlap between putative genes located within TEs. Note, that there are still TE insertions within introns of genes but rarely protein coding genes within annotated TEs.

p.9, l.188: "... TE mobilization": add reference

Done - we have added the following citations: SanMiguel et al. 1998 Nature Genetics, Jedlicka et al. 2020 Front. Plant Sci.

p.10, l/214: Superfamilies full name should be indicated in italics

Done

p.11 l.218-219: Isn't this expected by the cut and paste mechanism?

We are not convinced that this would be true. We actually are reporting more variable rates between superfamilies for TIR elements, some have higher rates and some are lower even though they are all cut and paste families. In contrast the class I families have quite similar rates to each other.

p.11 l.229: Change 'elements within a family' by 'copies within a TE family'
Done

p.14, l.279: Please precise the size range observed

It now reads

"These 'No TE SV's tended to be relatively small (min=51bp, median=155bp, max=340,969bp) and only accounted for 1.2% of the total Mb of SV sequence in B73."

p.14, l.281: "SV sequences that have partially (>95%) overlapping TE sequences": does this mean that 95% of the SV length is made of TE sequence or that 95% of the TE is within the SV? From the examples, it seems that it is the second case. Please clarify. Could cases of SV located

within larger TEs correspond to misannotated insertion within the TE rather than to deletions? To what extent could such insertions be missed in the annotation?

I think the reviewer might have mis-quoted this section of the manuscript. Partially is defined as < (less than), not > (greater than) 95%. This means that for any SV in this class there is some overlap with a TE but it accounts for less than 95% of the length of the annotated TE. This includes examples in which the SV includes an internal portion of an annotated TE or the SV includes one edge of an annotated TE. In both cases the SV is likely a deletion as it is only affecting a portion of a larger TE annotation.

p.15, l. 298: On average, 85% of the SVs is annotated as TE bases. This proportion is similar to this of TE sequences within the B73 genome. Could the authors comment on this? This could suggest that SV occur randomly in the genome rather than that TEs are major contributors to SVs.

This is an interesting question. While the B73v1 reported that 85% of the B73 genome is TE, there have been variable estimates depending on the TE annotation approaches that are used. In this work, we are using a filtered TE annotation that accounts for an average of 1.655Gb of the NAM genomes which have an average size of 2.132Gb. This means that the annotations we are using reflect a TE content of 77%. The observation that 85% of the SVs are annotated as TE reflect an enrichment for TEs relative to non-TE sequence but it also suggests that SVs include substantial sequence not-annotated as TEs. This is clarified in the revised text.

p.17, l.334: How was the 100-fold threshold chosen?

We initially plotted the relative SNP density across sliding windows of each chromosome. This identified clear regions of highly reduced diversity but we noted that these were never at a SNP density of zero, likely due to both sequence errors as well as spontaneous mutations. A 100-fold reduction in average rate is a relatively strict threshold that captures many but not all of the regions of reduced diversity.

p.17, l.339: Is this excepted considering the pedigree of the B73 inbred line? Where are these regions located? Are they shared between the NAM lines where they are found?

In general, yes. The lines that are more closely related to B73 or have potential common founder lines have substantially more SNP depleted regions while very few of the tropical lines have SNP depleted regions relative to B73. There are some overlapping regions of SNP-depleted regions that can be noted in Table S2.

p.17, l. 343: 'the SNP depleted regions': do they correspond to the 213 or to the subset presented above?

This refers to all of the SNP depleted regions, and we have amended the sentence to clarify this. "We expected to observe little variation across all SNP depleted regions given the relatively short divergence time between the two pairs of haplotypes."

p.18, l.356: why 224 and not 139?

There was a typo in this line (it should have been 218 not 224). This is 218 because it is referring to both the 139 TE-SV variants present in the B73 haplotype as well as the 79 TE=SV variants present in the NAM haplotypes (insertions could occur in either haplotype of these SNP depleted regions.

p.18, l.365: 'these regions': please precise which ones

We have amended the sentence to clarify that this refers to nearly 60% of all 'TE = SV' events across all SNP depleted regions.
"A per-family analysis revealed four TE families that account for > 10 'TE = SV' polymorphisms within the SNP depleted regions, and, in total, these four families accounted for nearly 60^ of all 'TE = SV' events in these SNP depleted regions (Figure 6A)."

p.18, l.376: 'sequence similarity': do the authors mean 'sequence identity'?

Yes, that is correct, we have changed the word accordingly.

p.21, l.428: Please cite previous work, such as this of Wang and Dooner, 2006 - PNAS

Good suggestion - we actually added this and the Brunner citation to a sentence just before this region to acknowledge this prior work.

p.22, l.454, l.457, l.458: please check typos and missing information

We apologize for the lack of numbers in these sentences. The appropriate numbers have now been added.

p.23, l.478: The authors could have proof of TE-related ectopic recombination using their data: what is the number of cases where the same TE family is found at the two borders, with breakpoint at the same site?

We likely do have some cases like this but they are not simple to quantify accurately. There are likely examples of ectopic recombination between related elements as well as recombination between LTRs resulting in solo LTRs.

p.23, l.480: The authors should also discuss the presence of deletions within the TE sequences. What is their proportion in the analyzed dataset?

We did not solely focus on deletions within a single TE. These are a portion of the Incomplete TE SV class (which also includes deletions that remove one side of a TE as well as flanking sequence. We are not sure why internal deletions of a TE are distinct from deletions that remove one side of a TE but not the other. Both reflect a deletion that removes a portion of a TE. Maybe we are missing something interesting here but we weren't convinced that internal TE deletions should be handled distinctly from deletions of one side of a TE.

p.24, l.507: Were long terminal repeat regions really removed?

No - sorry for the confusion. We are referring to the details of filtering the current output file from panEDTA. panEDTA outputs several distinct annotations for structurally annotated LTRs. In addition to a full length annotation, panEDTA also gives distinct annotations for the long terminal repeats and target site duplications. Consequently, we only retained the full length annotation and removed the additional annotations marking the long terminal repeats and target site duplications (if we did not do this we would be double counting bases as they would belong to multiple annotations). The full-length structural annotation we used in all downstream analyses still contain the long terminal repeat regions and target site duplications.

p.26, l.537: Please precise the fraction overlapping TE annotations

The gene annotations for the NAM genome sequences were filtered to remove TE related sequences as described in Hufford et al. We are not sure what the reviewer is looking for based on overlapping of TEs and genes. There are examples of TEs located within gene introns but there are very few examples of annotated genes located entirely within TEs.

p.26, l.554-556: For SVs that were found between B73 and several maize lines, were the breakpoints found always the same at the base level? This would help deciphering the accuracy of breakpoint prediction by AnchorWave.

Thanks for this suggestion. We used the example in Figure 4B that represents a common SV present in 14 lines. 12 of the 14 lines share identical coordinates for the SV (chr2:10901496_10902348). The other two lines have SV coordinates that are shifted one base pair (chr2:10901497_10902349). We added this information to the text.

Figure 1:
Precise the amount of overlap required for ambiguous cases

Thank you for noting that this was not clear. Anything classified as ambiguous does not meet the criteria for being shared (> 95% overlap with alignable sequence) or polymorphic (>95% overlap with structural variant sequence). This means that these ambiguous features can either entirely lie within unalignable regions or can lie across multiple region types without having 95% overlap with alignable or inserted sequence. We have added text to try and clarify this distinction.
"These ambiguous features included examples that were within unalignable regions as well as those that partially overlapped (<95% overlap) regions with alignable or inserted sequence (i.e., ambiguous features are those that do not meet the threshold to be either shared or polymorphic).

Figure 1 B: Add colors showing the amount corresponding to the different features (TE, gene syntenic, gene non syntenic).

It would be quite difficult to accurately visualize these features in this plot, especially for the

unalignable portion which is quite small already. However, we have include a table in the supplement (Table S4) that shows the proportions for each of these.

Figure 3A: Please add a statistical test

We are not sure what groups the reviewer would like to assess via a statistical test. We have not made any claims of significance within the manuscript. We could potentially test structural annotated TEs relative to homology annotated TEs or we could try to compare groups. We are not trying to make significant claims based on this figure but were rather simply describing polymorphic rates for different types of TEs.

Figure 3B: Please state how many families the >20 copies threshold leads to

Good suggestion, we have added this information to the manuscript.
"We limited this analysis to families with at least 20 members in B73 (N = 2,939 families)"

Figure 3C: The pattern observed is also likely impacted by the age of the copies. How does the pattern correlate with age of the copies? Is the age of the small families similar to this of families with larger copy number?

This is an intriguing comment and we agree that age is likely a factor. As the editor likely knows, it is possible to estimate the age for structurally intact LTR elements but much more difficult to generate age estimates for homology based annotations or DNA transposons. This means we can only get age estimates for <5% of the elements which substantially limits the power of an age-analysis, and this was why we had not included this work in the manuscript.

However, given the reviewers interest we can provide a summary of some analyses on age impacts here. We can take all B73 LTR families with greater than 20 elements. For each family, we can determine the average age of structurally annotated elements using LTR similarity as a proxy for age. Using the same age categories as Figure 2C (Category ~ Average Age: Very Young ~ 1.0, Young ~ [0.95,1.0), Moderate ~ [0.9,0.95), and Old - [0.8,0.9)), we find a marked excess of "Young" families (Very Young = 14, Young = 346, Moderate = 11, Old = 2). With this in mind, we recreate Figure 3C faceting by the average age of structurally annotated elements for the family (shown below). We find that "Very Young" families are restricted to smaller family sizes but exhibit a variable range of % polymorphic (~13 - 41%). "Young" families exhibit a distribution akin to Figure 3C, but this is driven by the overrepresentation of Young families in this analysis. "Moderate" families show a reduced range (~2.5 - 27%) of % polymorphic, but a somewhat broader range of family sizes. It is difficult to comment on the distribution of "Old" families as there are only 2 cases. The age of small families appears to belong mostly to "Very Young" and "Young" families, while the age of larger families appears to be either "Young", "Moderate", or "Old".

Given the small sample size of non-"Young" families, it is difficult to confidently claim whether there are correlations between the age of families and the percent of that family that is polymorphic. Generally, it does not seem like the age of the family is influencing the overall trends we observe.

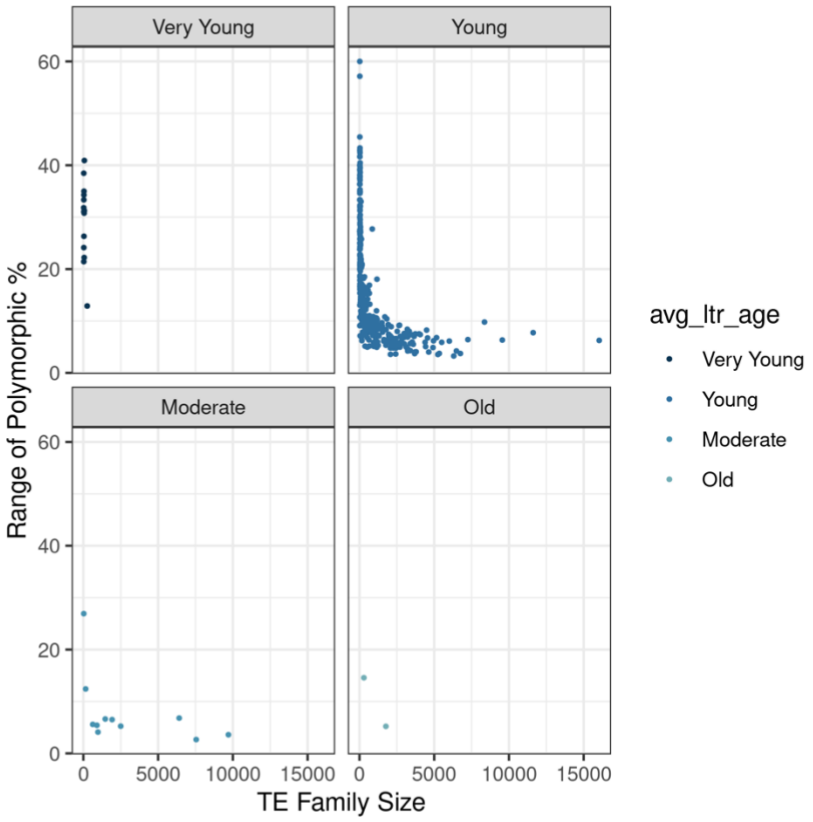

Reviewer's Responses to Questions

Comments to the Authors:

Please note here if the review is uploaded as an attachment.

Reviewer #1: In this work Munasinghe et colleagues analysed the independently assembled genome of several maize accessions. Interestingly, the analysis integrated structural variants and transposable elements (TEs) variations, allowing the integrated study of the plasticity of maize genome. Authors used regions with low SNPs accumulation to investigate recent structural variant events and managed to identify TE families with recent mobilisation in maize genome, likely occurred after domestication. This is a very interesting work which use a pan-genome comparison approach to reveal dynamics of maize genome plasticity. Authors concluded that many variants involving TE regions are likely events of deletions, while there is less evidence of TE new insertions. I believe these results are interesting and relevant to study how plant genomes evolve. One suggestion I have is related to the putative deletion events identified. Authors suggest that such variants could be the consequence of ectopic recombination events. So, I was wondering if authors have looked into a possible enrichment homology at the edges of these regions, which can be associated to non-homologous recombination events.

We thank the reviewer for the comments on the manuscript. Genome-wide, the comparisons of SVs and TEs showed the complex contributions of TEs to structural variation beyond simple insertion polymorphisms. It would be quite interesting to perform a genome-wide exploration to identify putative deletions that may be mediated by ectopic recombination. However, the long divergence time for many maize haplotypes can confound this analysis due to combinations of

nested TE insertions and mutations that reduce the accuracy of annotating precise TE boundaries. We could, however, explore sequence homology in our SNP depleted regions as they have less divergence time and can provide more insight.

We searched for potential signatures of LTRs or TIRs in the edges of the 'Multi TE SV' events. We did this by blasting the left edge of the SV against the right edge of the same SV. For LTRs, we took any SV 600bp. We then took the left 100bp and blasted it against the right 500bp. The longest alignment (>50bp) in the same orientation meant that SV was classified as a putative LTR insertion. For TIRs, we took any SV 100bp. We then took the left 50bp and blasted it against the right 50bp. The longest alignment (of any size) in the reverse orientation classified that SV as a putative TIR insertion. For any SVs that belong to both categories, we retain the classification derived from the longest alignment. Untested SVs were defined as an SV that was either (a) < 100bp in length (as it could not be tested as a TIR in our analysis) or (b) SVs between 100bp and 600bp long that were not classified as TIRs (as they were too short to be tested as an LTR). All remaining SVs were classified as putative deletion event (i.e., there was no blast hit). In the SNP depleted regions, we found that 84.5% of the Multi TE SV events have no evidence of either LTRs or TIRs (i.e., there were no blast hits between the edges of the SV). We find that 6.9% have evidence of TIRs (i.e., there is a blast alignment >50 bp in the and another 6.9% have evidence of LTRs. However, it is worth noting that we only required 10bp of direct or inverted homology so these might be over-estimates, especially for the LTRs. Genome wide, we see very similar numbers, 85.4% have no homology between the 5' end and the 3' end, while 3.9% and 6.7% have potential evidence for LTRs or TIRs (respectively). It is difficult to interpret these results in order to make generalizations about the frequency of ectopic recombination events, but we hope this analysis provides some insight into the observed homology at the edges of some 'Multi TE SV' events which may be mediated by ectopic recombination resulting in a deletion.

The remaining comments are quite minors, and are listed below:
Figure 1A. if B97 should be representative of any NAM line, please mention this in the legend, or use a general "NAM line" label in the figure panel.

We agree with the reviewer that it would make more sense in a general visual schematic to use "NAM" instead of a specific B73 line, and we have done so accordingly.

Figure 3A. it looks RIL TEs miss the structural annotation bar. Is this intentional?

Yes, it is intentional. There are no structurally annotated RILs. We have added a sentence to the figure caption to draw awareness to this as follows. "B73 TEs were grouped both by their assigned superfamily (x-axis) and by whether they were a structurally-annotated (yellow) or homology-annotated (orange) element. There are no structurally annotated RIL elements."

Line 101. Avoid nidified brackets

We have removed the interior parenthetical "(see method for details)" not only to avoid nidified brackets but also because it seemed unnecessary.

Line 162. The definition of syntenic and non-syntenic genes requires a better explanation. From method section, this appears to be done only in comparison to sorghum, but more information about the approach used is needed.

Yes - that is correct. We leveraged publicly available synteny calls generated from Hufford et al. 2021 (Science). We have added information from their supplement detailing how these assignments were generated.

"A full description of how synteny assignments were determined can be found in the Supplementary Materials (Fractionation Analysis) of Hufford et al. (2021). In summary, filtered exons from the outgroup Sorghum bicolor (Sbicolor_313_v3.1 from Phytozome) were aligned to the repeat masked NAM and B73 genomes. Orthologs were scored in each NAM line based on alignment of at least one Sorghum exon to a single gene-space locus syntenic with the query Sorghum gene. Orthologs could be classified as either fully retained, partial deletions,or fully fractionated. These results were then filtered for Sorghum exon alignments falling within the identified subgenome blocks of B73 version 4 associated with syntenic coordinates of Sorghum gene models. Gene density for each subgenome was determined by aligning the primary Sorghum CDS gene model sequences to the primary CDS gene model sequences of each NAM line. Syntenic relationships were then determined from these alignments."

Line 268-269. Did authors check that edges of the regions do not contain TIRs, TSD or homology, which could support transposition (of a misannotated TE) or maybe a recombination event?

Good suggestion. We carefully evaluated the sequences at the edges of this event and did not identify evidence for TSDs or TIRs (or extended homology). We mention this in the text now.

Line 298-300. Considering that maize genome contain >70% of transposons, this sentence is maybe excessive. I believe the finding that 85% of total SVs include TEs likely indicates an overrepresentation of TE DNA in variants, which if randomly distributed should be around 70% (nonetheless, a statistical test could be welcome here, especially considering that TEs are only associated to portion of "incomplete TE SVs").

This is a fair point and we have added text to this section to clarify. The analysis of B73 version 1 (Schnable et al 2009) reported 85% of the maize genome is annotated as TE. However, in the current analysis of the NAM genome and the TE annotations used in this study there is only 77% of the maize genome that is annotated as TEs. We do agree that the prior language was a bit excessive. We wanted to acknowledge in this section that while most SV sequence is TE-derived, this does not mean that most SVs reflect simple TE insertion polymorphisms. We have edited this section to better describe this concept.

Line 334. I think the use of ">20" is misleading? Can the five specific number of region be given in full?

We absolutely can and have added this information after each inbred line.

“In contrast, > 20 regions were identified between B73 and each of B97 (N = 22), Ky21 (N =27), MS71 (N = 21), Oh7B (N = 25), and Oh43 (N = 31).”

Line 341. Is 26 the number of regions or the reference to a citation? Can the precise number be given?

26 refers to the number of SNP-depleted regions that were 2Mb in size. We have amended this sentence to add clarity.
“While many (26 out of 213) of the regions were only 2Mb in length in B73, there were also 23 SNP depleted regions that were at least 10Mb in length.”

Line 454. Typo in “into”

Thank you for catching this error - it has been corrected in the revised manuscript.

457-458. what “X” stand for?

We thank the reviewer for catching this and have updated the sentence accordingly.
“In these regions, there are a total of 1.93 Mb of putative deletions (‘Incomplete TE SV’, ‘Multi TE SV’, and ‘TE Within SV’) and 1.14 Mb of putative TE insertions (‘TE = SV’ events).”

459-460. I believe an alternative possibility is that TE insertions have been underestimated (e.g. exclusion of Helitrons from the analysis).

That is a fair point - the exclusion of Helitrons could be an issue and we have now added that caveat. We do not think the exclusion of Helitrons would be an issue for the Incomplete TE SVs (since these occur within or overlapping a portion of an annotated TE). For the Multi TE SV and TE Within SV classes we are not sure how often Helitrons accumulate portions of other TEs. Prior studies have provided good evidence for accumulation of gene fragments within Helitrons but there is less data on collection of multiple TE fragments by helitrons.

Line 506. It is not explained why Helitrons (which are common TEs in maize genome) have been excluded from the analysis

Thank you for raising this issue. We realized that we had not clarified this in either the results section or in the methods section. Helitrons were excluded due to difficulties in generating high-quality annotations. While there are certainly good examples of helitrons that have been documented based on specific polymorphisms, it is quite difficult to generate genome-wide high quality annotations. Helitrons have very short structural features at the 5’ and 3’ ends that are not particularly diagnostic and the fact that helitrons often capture non-TE sequences complicates homology assessments. In a prior study (Anderson et al., 2019), we found that genome-wide helitron annotations had >1000 examples in which a large helitron encompassed a syntenic gene. The fact that the gene was syntenic with other grassess (where it was not annotated within a TE) suggested it had not moved and that these were false positive helitron annotations. In contrast, it was exceedingly rare for the same annotation tools to predict syntenic

genes within TIR or LTR transposons. We have added short text clarifying this rationale to both the results and methods sections.

Line 507. Typo in "duplications"

Done

Line 508. Can you replace "handful" with a precise quantification?

Done – we have removed the word "handful" and replaced it with the phrase "small percentage" as it is more accurate, and we have now added the median number of duplicate annotations (as well as the % of duplicate annotations of the total) at the end of the sentence.

"We removed a small percentage of 'duplicate' annotations with different IDs but identical coordinate positions, superfamily classifications, and family classifications (median of N = 418 annotations, approximately 0.037%, removed per NAM line)."

The list of annotated TEs and structural variants should be made available as supplementary material. I believe the authors will also benefit in providing with their manuscript a .gff file containing the positions of the structural variants detected annotated on the B73 genome, as well as the TE annotation, because other researchers could directly use them in future genome comparison studies.

We agree with the reviewer that several of the datasets referenced in our manuscript would be of use to the community and should, therefore, be made publicly available. We would like to note, however, that some of these datasets are already available. We used TE annotations generated using panEDTA (developed by Ou et al. in Review). While this manuscript is currently under review, the TE annotation files are publicly available on maizeGDB (https://maizegdb.org/NAM_project). We performed filtering on the full list of annotated TEs to generate a reduced set of TE annotations that we had much higher confidence in. This set of TE annotations, as well as their status as either shared/polymorphic/ambiguous, has been uploaded to Dryad as part of the Data Availability of this manuscript. It occurred to us that it may not be clear from our manuscript that this full set of annotations is publicly available, and we have added a sentence to the methods highlighting this.

"These annotations are publicly available for download through MaizeGDB (https://maizegdb.org/NAM_project)."

Similarly, while we provided the AnchorWave GVCFs which we parsed to call structural variants, we acknowledge that the location of these structural variants may be of use to the community. We have consequently generated these files in bed format (we did not use gff since there is no strand/score information) and have uploaded these files to Dryad.

Reviewer #2:

The manuscript submitted by Munasinghe et al. describes a comparative genomic analysis of several well assembled maize genome to study the TE-driven genomic differenciation in this crop species.
This paper is well written (apart from few lines in the discussion...) and the analyses are well described and I have no problem with most interpretations of the results.
The authors have used a whole genome alignment method (Anchorwave) combined with an annotation of TEs of the genome of 26 NAM lines (panEDTA) to identify Transposable element insertion polymorphisms (TIPs). Using this approach, they found that 30 % of all TEs are polymorphic between B73 and the NAMs. A thorough analysis of this data revealed several features of TE-driven genomic changes in maize that are highly relevant in the field of plant evolutionary genomics.
1- the authors clearly show that only 10% of the SVs could be accounted for by a transposition event (SV = TE), while the rest of the variants encompass several TEs and suggest that they are the result of TE elimination which leads the authors to conclude that maize genome is decreasing in size through this TE elimination process.
2- Through an elegant method consisting in targetting low SNP regions, the authors identify young insertions and therefore putatively recently active TE families.
3- the analysis of SVs in genes show that non syntenic genes (ie genes that are not conserved across grass genomes) have a higher propensity to be deleted (or inserted).
I have no major objection with the publication of this paper as it stands given only minor modifications are made :

We thank the reviewer for their careful reading of the manuscript and for their feedback. We have addressed the reviewer's concerns below.

-l 102 change "transposons" by "transposable elements"

We have changed the following sentences by swapping in transposable elements for transposons here.
"After numerous standard quality control steps (e.g., excluding helitron annotations, non-TE repeats, and potentially misannotated TEs (see methods for details)), we found that, on average NAM lines had 858,902 transposable elements. 7.9% of these TEs were structurally annotated and the remaining 92.1% relied on homology-based annotations (Table S1). The average total of transposable element sequence in the NAM genomes was 1,655Mb (min=1,634Mb, max =1,673Mb) with structurally annotated TEs accounting for an average of 31.2% of the total TE Mb (Table S1).

-l 234 -> what does "constrained proportion" mean ? please rephrase.

We have added context for that sentence that should explain what we mean by constrained (i.e,, the percent polymorphic is capped at roughly 20%).
"However, the larger families (> 1000 copies, N = 168) tended to have a constrained proportion of polymorphic TEs, capped at 20% polymorphic (Figure 3C).

-l 218-224 -> I don't think one could draw any conclusion on the fact that some class II families exhibit more polymorphisms than others in the 26 NAMs. It just depends on whether a genome

bears an active copy. The fact that monomorphic elements are still present in the genome strongly suggests that the corresponding family is still active, otherwise it would have been eliminated from the genome.

We included information on differences between superfamilies for context, but we agree with the reviewer that one should be cautious when drawing conclusions from these results. We do comment that we see more variation across retrotransposon families relative to DNA transposons, but we also mentioned that this trend could be either biological or artefactual. We have added a sentence to the end of that paragraph explicitly urging readers to exercise caution. "We encourage exercising caution when drawing conclusions from differences in polymorphic TE proportions. It is unclear whether these differences were due to biological differences among superfamilies or technical artifacts such as differences in size or annotations."

l 334-342 I was missing some information of the genetic relatedness between the NAMs. Low SNP regions suggest a common recent ancestry. But has this been quantified at some point?

Good question. The NAM lines were selected to maximize allelic diversity (based on SSR data available for a panel in ~2005. While many of the NAM lines are of tropical origin and have little or no pedigree relationship to B73, there are also several non-stiff stalk lines which are derived from synthetic populations. Some of the parents of these synthetic populations were also parents of the B73 synthetic population and, therefore, there can be regions of shared sequence. We are very purposeful in our manuscript to avoid using the term identity by descent, which would infer descent from a common ancestor. Instead, we refer to these regions as identical by state. We selected our 100-fold lower SNP rate based on comparison to the genome-wide SNP rate as it was sufficiently lower than genome-wide patterns.

L 455-460 I think that the authors did not review this page of the manuscript before submission….."X Mb of putative…"

We thank the reviewer for catching this and have updated the sentence accordingly.
"In these regions, there are a total of 1.93 Mb of putative deletions ('Incomplete TE SV', 'Multi TE SV', and 'TE Within SV') and 1.14 Mb of putative TE insertions ('TE = SV' events)."
