# Supplementary material for: Combined analysis of transposable elements and structural variation in maize genomes reveals genome contraction outpaces expansion
Source: PLoS Genet. 2023 Dec 22;19(12):e1011086. doi: 10.1371/journal.pgen.1011086 (PMC10773942; doi:10.1371/journal.pgen.1011086)
Supplement: Attachment — Submitted filename: ResponseToReviewers_Munasinghe_etal_11.20.23.docx [file pgen.1011086.s014.docx]

**Response to reviewers - Munasinghe et al.**

Overall Response: We appreciate the comments of the editor and the reviewers. We have worked to revise the manuscript in light of these comments, and we discuss each one below

**Editor Comments**

*In particular, after reviewing the data available, I pointed out that in the ‘Polymorphic TE calls’ files available on Dryad, there is no column classifying the TE copies as used in the manuscript: column 18 classifies TEs based on “shared, polymorphic, or ambiguous”, but there is no column with the ‘TE=SV, multiTE, etc.’ classification. Since the authors have this information, and since they are the data used to draw conclusions of the article, it would be nice that they share it with the community.*

We agree with the editor that this information should be shared with the community, but, it is our opinion, that the ‘polymorphic_TE_calls’ files on Dryad may not be the most appropriate file to include this information in. Only structural variants were categorized as either ‘No TE SV’, ‘Incomplete TE SV’, ‘TE = SV’, ‘Multi TE SV’, or ‘TE Within SV’, but the ‘polymorphic_TE_calls’ files lists the calls for all TEs (either shared, polymorphic, ambiguous) which may overlap other alignable or unalignable regions as well. This would result in many of the entries in that column being ‘NA’.

Consequently, we have provided an additional data file that can easily be linked to the information stored in the ‘polymorphic_TE_calls’. This data file “X_Structural_Variant_Category_Info.tsv’ lists out every SV, their genomic position, their category assignment, and which TEs they overlap. There is a B73 version, for all SVs with sequence in B73, and a NAM version, for all SVs with sequence in a given NAM line. Anyone in the community can now select for their SVs of interest, get its category assignment, and obtain the TEs it overlaps. They can link the TE name in this file to the TE in the ‘polymorphic_TE_calls’ files to get detailed information about the TE.

*Besides this, I have a few minor points, mainly corresponding to typos:*

*p.2, l.24 : change ‘de-novo’ to ‘de novo’ in italics*

Done

*p.3, l.2: The wording ‘pays a role’ is misleading. The authors find that the major process underlying structural variation is deletions. In this case, TEs are not actively involved in generating the diversity observed. Rather, they are passive elements on which the deletion process occurs. Please modify.*

We think this refers to p.3, l.52, and, if so, we agree with the editor on this comment. We have changed this sentence accordingly.

Page 3, lines 52 – 54:

“Our results highlight the complex relationship between transposable elements and genomic diversity, and we provide a valuable resource for future studies of diversity across transposon-rich maize genomes.”

*p.6, l.114-123. As pointed by reviewer in the original version, ‘transposon’ should be replaced by ‘transposable elements’ for more clarity.*

We are not 100% sure if the lines referenced are accurate, as the revised version of the manuscript does use the word transposon throughout this paragraph. If there are additional places in the manuscript where the editor feels we should change transposable element to transposon, we are open to doing so.

*p.11, l.217: vs should be in italics*

Done

*p.15, l. 294: i.e. should be in italics*

We use *i.e.* several places throughout the manuscript, and we have updated all of them to be italicized

*p.19, l.396 and l.397: this should be 139+79=218 and not 224. Please correct the typo.*

Done.

*p.22, l.452: via should be in italics*

Done.

**Reviewer #1:**

*Authors provided a revised version of their manuscript which fulfills my requests. I do not have further comments to be addressed.*

**Reviewer #2:**

*The authors have satisfactorily addressed the minor points I made in my first review.*

We thank both Reviewer #1 and #2 for their time and consideration of our manuscript.
